# Gray and White Matter Changes in Subjective Cognitive Impairment, Amnestic Mild Cognitive Impairment and Alzheimer's Disease: A Voxel-Based Analysis Study

**DOI:** 10.1371/journal.pone.0104007

**Published:** 2014-08-05

**Authors:** Kuniaki Kiuchi, Soichiro Kitamura, Toshiaki Taoka, Fumihiko Yasuno, Masami Tanimura, Kiwamu Matsuoka, Daisuke Ikawa, Michihiro Toritsuka, Kazumichi Hashimoto, Manabu Makinodan, Jun Kosaka, Masayuki Morikawa, Kimihiko Kichikawa, Toshifumi Kishimoto

**Affiliations:** 1 Department of Psychiatry, Nara Medical University, Kashihara, Japan; 2 Medical Center for Dementia, Nara Medical University, Kashihara, Japan; 3 Sakai City Mental Health Center, Sakai, Japan; 4 Molecular Imaging Center, National Institute of Radiological Sciences, Chiba, Japan; 5 Department of Radiology, Nara Medical University, Kashihara, Japan; 6 Faculty of Psychology, Doshisha University, Kyoto, Japan; 7 Mie Prefectural Mental Care Center, Tsu, Japan; University of California, San Francisco, United States of America

## Abstract

Subjective cognitive impairment may be a very early at-risk period of the continuum of dementia. However, it is difficult to discriminate at-risk states from normal aging. Thus, detection of the early pathological changes in the subjective cognitive impairment period is needed. To elucidate these changes, we employed diffusion tensor imaging and volumetry analysis, and compared subjective cognitive impairment with normal, mild cognitive impairment and Alzheimer's disease. The subjects in this study were 39 Alzheimer's disease, 43 mild cognitive impairment, 28 subjective cognitive impairment and 41 normal controls. There were no statistically significant differences between the normal control and subjective cognitive impairment groups in all measures. Alzheimer's disease and mild cognitive impairment had the same extent of brain atrophy and diffusion changes. These results are consistent with the hypothetical model of the dynamic biomarkers of Alzheimer's disease.

## Introduction

The relationship between subjective and objective memory impairment is more complicated in older adults. Around 25–56% of older adults have subjective memory complaints, and such complaints may be a predictor of future dementia [1]. Subjective cognitive impairment (SCI) may be observed prior to amnestic mild cognitive impairment (MCI) in the continuum of the disease progression [1]–[4]. Amnestic MCI is an at-risk period of dementia, converting to dementia at a rate of 10–15% per year [5]–[12]. About 7–8% of healthy older people with SCI progress to MCI or convert to dementia every year. [3] Jessen et al. reported that subjective memory impairment (SMI) subjects with worry showed a greater risk for conversion to Alzheimer's disease (AD) than did SMI subjects without worry or subjects without SMI [4]. AD is the most common type of neurodegenerative disorder, and its main clinical feature is memory impairment with impaired awareness even in the earliest stages [13]. Galeone et al. reported that AD and MCI subjects showed reduced awareness of memory difficulties and significant memory monitoring deficits [14]. Additionally, the insight into memory impairment becomes weaker with disease progression, a process that may be used to predict conversion from MCI to AD. Therefore, the self-awareness of poor memory function is considered to be a very early change in the preclinical stage of AD. In AD patients and MCI subjects, amyloid β (Aβ)-plaque biomarkers have largely reached a plateau by the time clinical symptoms appear [15]. Similarly, genetic at-risk individuals demonstrate Aβ accumulation many years before the onset of impaired cognitive function [16]–[18]. Thus, those displaying SCI may already have some brain pathological abnormalities. Identifying the prodromal stage of AD is a major target for clinical research and disease-modifying therapies [19], [20]. At a more basic level, we should discriminate at-risk from normal aged adults with less expensive tools. Although biomarkers for AD are now available even at the preclinical stage, their acquisition is invasive and/or costly for patients. (e.g., lumbar puncture, positron emission tomography (PET) scan). Neuroimaging studies of SCI have shown abnormalities of Aβ deposition, diffusion tensor imaging (DTI) and grey matter volume. Selnes et al. reported that DTI surpasses cerebrospinal fluid as a predictor of cognitive decline and brain atrophy in SCI subjects [21]. In a PET study, high Aβ deposition in older adults was associated with future cognitive decline [22]. SCI subjects also showed decreased volumes of medial temporal lobe structures compared to subjects without subjective cognitive failure [23], [24]. However, there have been few imaging studies on SCI, possibly because of the difficulty in recruiting and assessing SCI subjects. Even in the reported studies, the number of subjects was relatively small. In addition, most of the previous SCI studies did not use more complicated memory tests such as the logical memory II subscale from the Wechsler memory scale; hence, those imaging studies might have contained MCI subjects. The proposed Reisberg criteria for primary idiopathic subjective cognitive impairment [3] are as follows: (1) Presence of subjective cognitive deficits; (2) Belief that one's cognitive capacities have declined in comparison with 5 or 10 years previously; (3) Absence of significant medical, neurologic, or psychiatric conditions; (4) Absence of overt cognitive deficits; (5) Cognitive performance in a general normal range; (6) Absence of dementia. In this study, we screened SCI subjects who met these criteria, except (2), and who further visited our hospital for consultation because of memory deficit. The reason for the exclusion of criterion (2) was that subjects did not undergo cognitive tests in the previous 5 or 10 years. In the current imaging study, we examined the differences in DTI indices and cortical atrophy by comparing AD, MCI, SCI and normal controls (NC). We proposed that DTI or 3D-MRI would be useful as an early stage biomarker. Briefly, our hypothesis was as follows:

1. Compared with NC, we will observe diffusion changes in AD, MCI, and SCI subjects.
2. By comparing NC, we can observe volumetric changes among AD, MCI, and SCI subjects.
3. These SCI changes would be useful for prediction of preclinical stages in the continuum of AD.

## Method

### 2.1. Subjects

The subjects in this study were 39 (11 males and 28 females) mild to moderate patients with probable AD, 43 (6 males and 37 females) amnestic MCI patients and 28 (9 males and 19 females) SCI individuals, recruited from the Department of Psychiatry, Nara Medical University, Kashihara, Japan. NCs were recruited from local resident associations and elderly clubs in Kashihara city. Sixty NCs underwent a medical examination and cognitive assessment, and 19 of them were excluded for meeting the criteria for MCI, probable AD, DSM-IV axis I disorder or the exclusion criteria mentioned below. Probable AD was diagnosed according to the National Institute of Neurological and Communicative Disorders and Stroke and the Alzheimer's Disease and Related Disorders Association criteria [25]. Amnestic MCI was diagnosed according to the criteria defined by Petersen [26], [27]: subjective or informed-by-partner memory complaints confirmed by impaired memory function (scoring below the education-adjusted cutoff on the logical memory II subscale from the Wechsler memory scale; WMS-R LM II), a Mini Mental State Examination (MMSE) score greater than 23, absence of significant levels of impairment in other cognitive domains, and essentially preserved activities of daily living. The education-adjusted cutoff scores of WMS-R LM II are as follows: a) education years ≥16, LMII score ≤8; b) education years 10 to 15, LMII score ≤4; c) education years 0 to 9, LMII score ≤2.

The SCI subjects had become aware of poor memory function and came to our hospital for consultation. To be classified as SCI, the subjects had normal memory function on WMS-R LM II and scores above cut-off on MMSE. Three SCI subjects had converted to amnestic MCI and one to AD by the conclusion of this study. This study was approved by the Ethics Review Board of Nara Medical University. Written informed consent was obtained from each of the subjects prior to their participation.

A somatic and neurological evaluation was performed in all subjects, with a routine laboratory examination and brain structural MRI. Exclusion criteria for all subjects were: a history of substantial head injury, seizures, neurological diseases, impaired thyroid function, and corticosteroid use. Cerebral white matter hyperintensities on T2-weighted images were rated for all participants using the deep white-matter hyperintensity (DWMH) grade of the Fazekas scale [28]. Subjects with cortical infarctions or DWMH grade 3 or 4 on T2-weighted images were excluded, whereas subjects with small lacunae in white matter (fewer than 5 lesions on T2-weighted images) were included. All participants were screened for comorbid medical and psychiatric conditions by means of clinical, physical, and neurological examinations. Cognitive function was assessed according to a standardized cognitive battery of tests, including MMSE and the Alzheimer's disease assessment scale-cognitive component (ADAS-Cog). Global deterioration scale (GDS) stage was also determined by clinical interview [29], [30]. NC is thought to be the first stage of the GDS scale, SCI the second stage, MCI the third, and AD patients were placed in the fourth and fifth stages.

### 2.2. Cognitive assessment

Assessment of cognitive function was carried out according to a standardized battery of tests, including the MMSE, WMS-R LM II (story A) and the ADAS-Cog. NCs did not undertake the ADAS-Cog. Two well-trained psychologists evaluated the cognitive functions of all subjects.

### 2.3. Data acquisition by MRI

All MRI examinations were performed using a 1.5-Tesla scanner (Magnetom Sonata, Siemens AG, Erlangen, Germany). DT images were acquired with echo-planar imaging (EPI) sequence (b = 0 and 1000 s/mm^2^, repetition time (TR)  = 4900 ms, echo time (TE)  = 85 ms, field of view (FOV)  = 230 mm, matrix  = 128×128, slice thickness 3 mm without gap, number of averages  = 6; 50 contiguous slice images; acquisition time, 6 minutes). The reconstruction matrix was 256×256 by interpolation, and 2×2×2 mm voxel data were obtained. Motion probing gradient (MPG) was applied in 6 directions. High-resolution three-dimensional T1-weighted images were acquired using a magnetization prepared rapid gradient echo (MPRAGE) sequence (TR  = 1500 ms, TE  = 3.93 ms, inversion time (TI)  = 800 ms, flip angle  = 15°, FOV  = 233×233 mm, slice thickness  = 1.25 mm; 144 sections in the sagittal plane; acquisition matrix, 256×256; acquired resolution, 1×1×1 mm). We also acquired T1-weighted (spin-echo; TR  = 500, TE  = 20) and T2-weighted (turbo spin-echo; TR  = 4000, TE  = 110) images.

### 2.4. MRI processing and voxel-based analysis (VBA)

The obtained diffusion images were visually inspected for apparent artifacts by a radiologist. Automated image preprocessing and statistical analysis were carried out using statistical parametrical mapping software (SPM8, Wellcome Department of Imaging Neuroscience, London, UK) running in MATLAB (MathWorks, Natick, MA, USA). For each subject, distortions induced by eddy currents and head motion were corrected by affine registration of the diffusion images to the non-diffusion weighted images (b value  = 0 s/mm^2^).

A brain mask of each subject was created using the Brain Extraction Toolbox (BET). The diffusion tensor indices of each voxel were calculated by FMRI's Diffusion Toolbox (FDT), and then the mean diffusivity (MD) and fractional anisotropy (FA) maps were generated for each subject. We coregistered the individual T1-MPRAGE images to the B0 map, and then normalized the T1-MPRAGE images into the standard MNI space and applied the transformation matrix to normalize the generated FA and MD images. Images were shown at a final voxel size of 2×2×2 mm resolution. The resulting transformation was then applied to the MD map for spatial normalization. The normalized maps were spatially smoothed with a 6-mm isotropic Gaussian filter.

Gray matter image preprocessing and statistical analyses were also carried out using SPM8 software (Wellcome Department of Imaging Neuroscience Group, London, UK; http://www.fil.ion.ucl.ac.uk/spm), in which we applied VBM implemented in the VBM8 toolbox (http://dbm.neuro.uni-jena.de/vbm.html) with default parameters. Images were bias-corrected, tissue-classified, and registered using linear (12-parameter affine) and non-linear transformations (warping), within a unified model [31]. Subsequently, analyses were performed on gray matter (GM) segments, which were multiplied by the non-linear components derived from the normalization matrix in order to preserve actual GM values locally (modulated GM volumes). Importantly, the segments were not multiplied by the linear components of the registration in order to account for individual differences in brain orientation, alignment, and size globally. Finally, the modulated volumes were smoothed with a Gaussian kernel of 6 mm full width at half maximum (FWHM).

Normalized and smoothed FA, MD and GM image maps were compared with voxel-based analysis among the four subject groups. Statistical inferences were made at a voxel-level threshold of p<0.001, uncorrected, with a minimum cluster size of 30 voxels. Fazekas DWMH grade, age, gender, education and cholinesterase inhibitor use were treated as covariant components.

### 2.5. Statistical analysis

Demographic data were analyzed using the Statistical Package for Social Sciences (SPSS for Windows, Version 16.0; SPSS, Chicago, IL). We performed the χ^2^ test of differences in gender distribution and Fazekas DWMH score across the groups and used the Kruskal–Wallis test to evaluate systematic differences in age and education across groups.

## Results

### 3.1. Demographic Data

There were significant differences in gender, age and cholinesterase inhibitor administration across the groups. Seventeen AD patients, 16 MCI patients and 1 SCI subject were taking cholinesterase inhibitors at the time of MRI acquisition and cognitive function assessment. As mentioned earlier, GDS stages were also categorized. AD patients were placed in the fourth or fifth stage, with 25 mild AD patients classified as fourth stage and 14 moderate AD patients as fifth stage. The mean MMSE score was 20.8±2.1 for AD subjects, 26.3±1.5 for MCI subjects, 28.5±1.5 for SCI subjects, and 28.9±1.6 for controls (Table 1).

**Table 1:** Demographic and diagnostic data of the participants.

	NC	SCI	MCI	AD	
Number (n)	41	28	43	39	*p*
Gender, f/m	23/18	19/9	37/6	28/11	<.05*
GDS stage	1	2	3	4 or 5**	
Age, mean (SD), y	75.2 (5.34)	70.5 (7.30)	74.6 (6.40)	73.2 (7.98)	<.05***
Education, mean (SD), y	12.27 (2.05)	12.36 (2.23)	11.56 (2.45)	11.85 (2.38)	N.S.***
MMSE, mean (SD)	28.9 (1.55)	28.5 (1.50)	26.3 (1.47)	20.8 (2.11)	-
ADAS-Cog., mean (SD)	-	5.48 (2.18)****	11.23 (3.96)	17.00 (5.63)	-
WMS-R					
Logical Memory I A	10.76 (2.85)	11.86 (3.84)	3.53 (2.35)	2.26 (0.18)	-
II A	10.20 (3.30)	9.18 (3.56)	1.02 (1.35)	0.18 (0.56)	-
Fazekas DWMH grade					
0	17	11	13	17	N.S.*
1	22	15	22	17	N.S.*
2	2	2	8	5	N.S.*
ChEI administration	0	2	16	17	<.0001*

NC, normal controls; SCI, subjective cognitive impairment; MCI, mild cognitive impairment; AD, Alzheimer's disease; GDS, Global Deterioration Scale; MMSE, Mini Mental State Examination; ADAS-Cog., Alzheimer's disease assessment scale - cognitive subscale; WMS-R, Wechsler memory scale - revised; N.S., not significant; DWMH, deep white matter hyperintensity; ChEI, cholinesterase inhibitor.

*χ^2^ test, **GDS; stage 4, n = 25, stage 5, n = 14, ***one way analysis of variance, ****lack of one subject's data.

### 3.2. Voxel-based analysis

**Figure 1:**
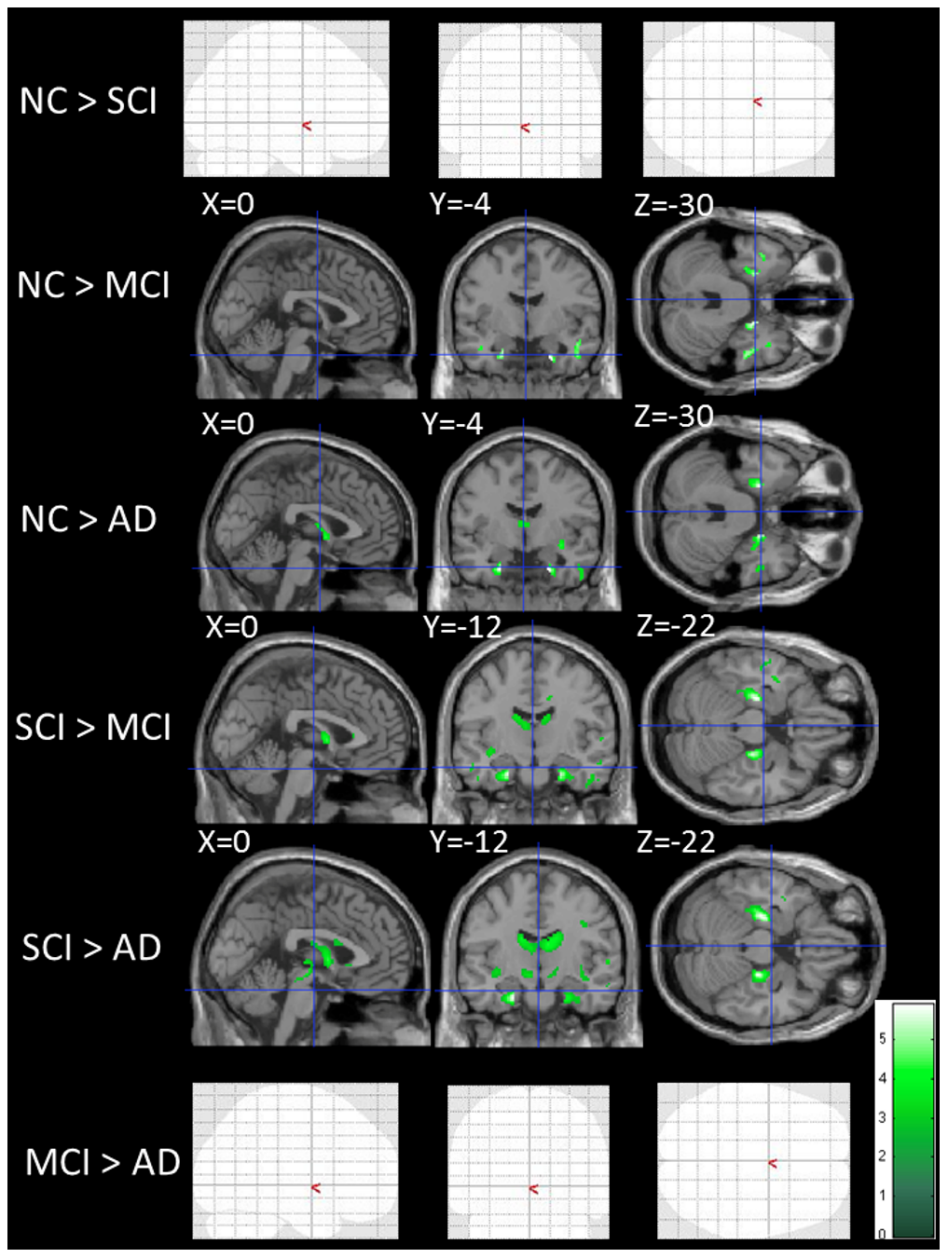
Differences in fractional anisotropy among normal controls, subjective cognitive impairment, mild cognitive impairment and Alzheimer's disease. NC indicates normal control; SCI, subjective cognitive impairment; MCI, mild cognitive impairment; AD, Alzheimer's disease. The statistical brain maps show colored voxels (green to light green) in regions of significantly lower fractional anisotropy (FA) (p<0.001). The blank brain maps (NC > SCI, MCI > AD) show that there are no significant differences between those subject groups (NC > SCI, MCI > AD).

**Figure 2:**
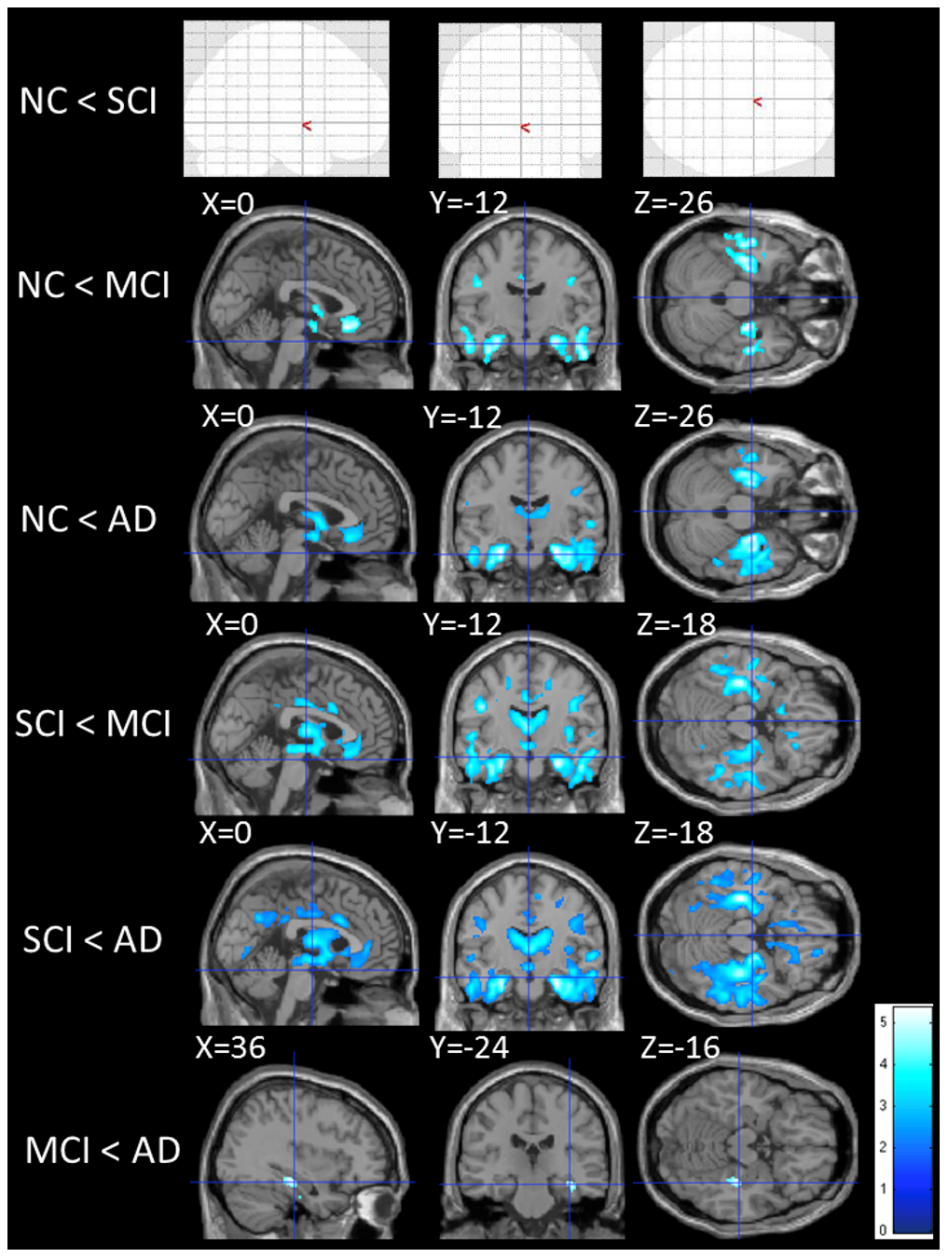
Group differences in mean diffusivity. The statistical brain maps show colored voxels (dark blue to light blue) in regions of significantly higher mean diffusivity (MD) (p<0.001). The blank brain map (NC < SCI) shows that there are no significant differences between NC and SCI groups.

**Figure 3:**
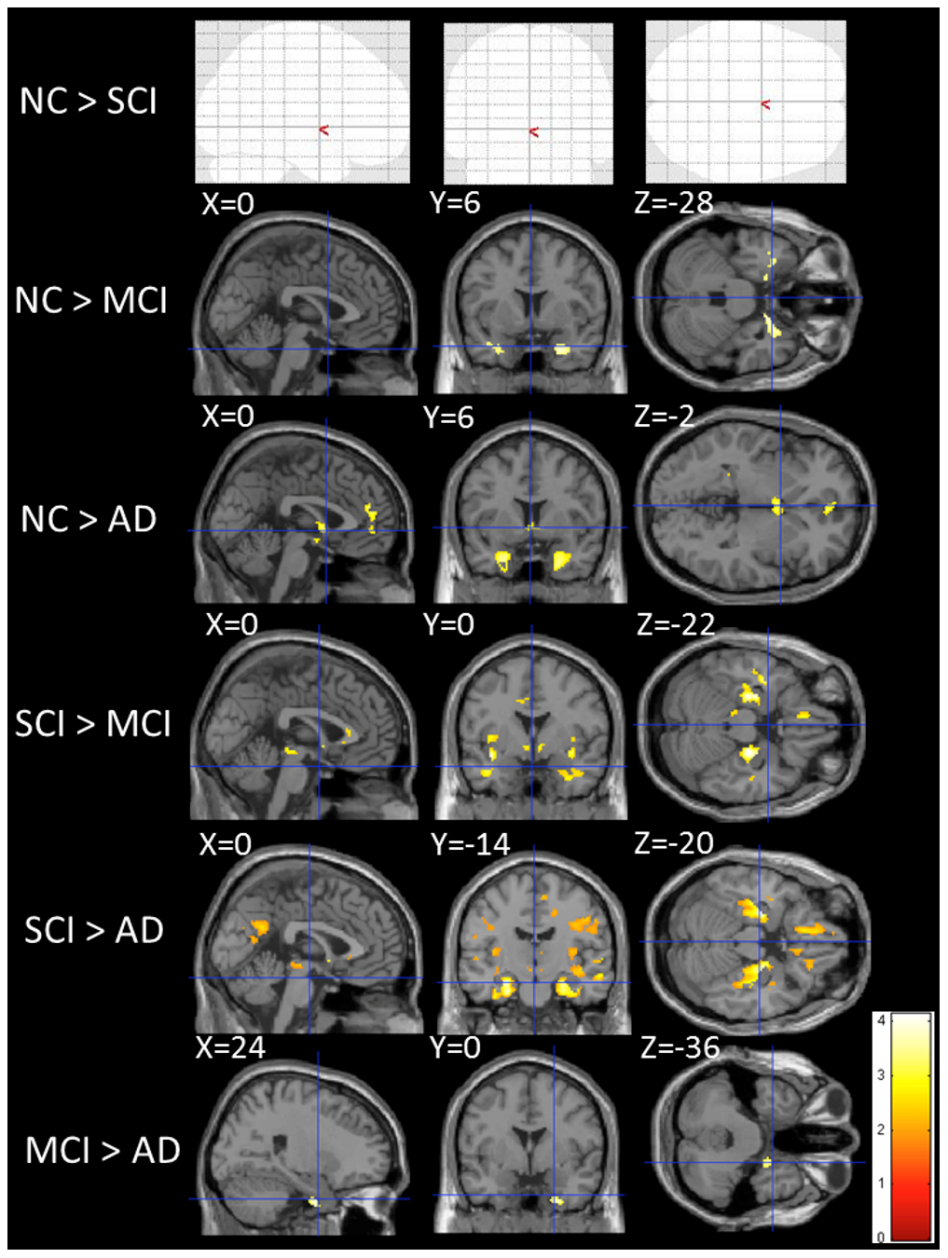
Gray matter atrophy across subjects groups. Voxel-based analysis showing regions of gray matter atrophy across groups. Clusters are overlaid on the MNI standard brain. Red- to yellow-colored voxels show regions with significance in the analyses. The blank brain map (NC > SCI) shows that there are no significant differences between NC and SCI groups.

Imaging results for all comparison groups are shown in Tables S1–S3 and Figs. 1–3. FA and MD are diffusion tensor imaging markers commonly used in the study of microstructural white matter abnormalities in many pathological states. It is believed that factors such as axonal density, myelination and homogeneity in the axonal orientation affect the degree of these diffusion markers. Compared with NC, MCI and AD groups showed significantly lower FA in the medial temporal region. Similarly, compared with SCI, MCI and AD groups had significantly lower FA in the medial temporal region and deep white matter (Fig. 1 and Table S1). There were no statistically significant differences between the AD and MCI groups.

As for MD measures, AD and MCI had more widespread, higher MD regions mainly in the temporal lobe and cingulum than NC. Compared with SCI, AD and MCI had elevated MD in the temporal lobe, frontal lobe and precuneus (Fig. 2 and Table S2). There were widespread, significant differences in GM volume in NC compared with AD and MCI. AD and MCI showed atrophic changes in the medial temporal lobe and frontal lobe relative to NC. Similarly, the AD and MCI groups had more prominently atrophic regions than the SCI group in the frontal lobe, temporal lobe, cingulate gyrus and precuneus of the superior parietal lobe (Fig. 3 and Table S3).

There were no statistically significant differences between the NC and SCI groups in all measures (Tables S2–3, Figs. 1–3).

Additionally, both AD and MCI had the same extent of diffusion changes. To assess the effect of medial temporal volume atrophy on the results of the voxel-based DTI analysis of the AD and MCI groups, voxel-wise FA and MD differences between these groups were examined by adding the medial temporal volume as a covariate. The medial temporal volume of each subject was calculated by averaging the values for all voxels within the spherical volume of interest (VOI) placed on the region where the AD group showed significant atrophic change when compared to the MCI group (Fig. 3). The center of the spherical VOI (5-mm radius) was determined from the MNI coordinates with peak t-value (coordinate X = 25, Y = −2, Z = −32; see Table S3). The results showed that, taking into account the medial temporal atrophy, there were no significant differences of FA and MD in the medial temporal regions between the AD and MCI groups (Fig. S1–2).

## Discussion

In our study, the AD and MCI groups showed more widespread abnormal regions than the NC and SCI groups. AD and MCI showed almost the same degree of abnormalities in white and gray matter. However, there were no statistically significant differences between the SCI and NC groups by any of the measures. These results suggest that it may be difficult to investigate microstructural changes of the SCI stage using structural or diffusion tensor MRI. In addition, 3 SCI subjects converted to amnestic MCI and one subject converted to AD after this study. Therefore, some of the SCI subjects in this study may have undergone some pathological changes (e.g., deposition of amyloid beta). Briefly, it may be difficult to show initial changes at the SCI stage by structural MRI or DTI. In addition, this speculation would be consistent with the preclinical stage of the hypothetical model of dynamic biomarkers of Alzheimer's disease [15], [32]. The model is based on the hypothesis that amyloid-β accumulation is an upstream event in the cascade resulting in synaptic dysfunction, which may in turn trigger neurodegeneration and cell loss causing structural changes in the brain. Finally, the full pathologic cascade of events results in dementia. Similarly, the current results also suggested that the AD and MCI groups had almost the same extent of pathological changes, which is also consistent with the hypothetical model. In addition, this result is consistent with our previous tractography-based DTI study on AD and MCI [33].

Three studies have reported volume reduction in temporal lobe structures in subjects with subjective memory or cognitive impairment. One study showed that subjective memory impairment subjects had smaller medial temporal structures [24]. However, as measurement was performed by manual outlining and only one rater, the results might have suffered from bias [34]–[36]. Although the other two studies also showed brain atrophies in SCI, cognitive testing was insufficient, suggesting that both studies might have included MCI subjects as SCI subjects [37], [23]. These two studies did not use specific memory tests such as the logical memory II subtest of the Wechsler memory scale. Without such a test, it is difficult to diagnose between SCI and amnestic MCI [38], [39].

To our knowledge, five DTI studies on SCI have been reported [21], [40]–[43]. Four out of the five studies were conducted by the same research group [21], [40]–[42]. Three of these studies combined cerebrospinal fluid (CSF) biomarkers and DTI [21], [41], [42], and two of them showed significant diffusion changes in cingulum fiber [41], [42]. However, because of the small number of SCI subjects in two of these studies, the SCI and MCI subjects were combined and the MCI subjects' results were mainly shown [41], [42]. They also showed that DTI parameters were a better predictor of disease progression than CSF biomarkers in SCI, and 5 of 11 SCI subjects converted to AD during the follow-up period, as did 6 of 43 MCI patients [21]. Because the SCI subjects have high rate conversion to AD relative to the MCI subjects, the subjects in that study may be highly-selected and have been far from ideal SCI subjects. However, combining CSF biomarkers and imaging results would be ideal because SCI subjects would likely show very few structural imaging changes.

Meanwhile, Wang et al. reported that a voxel-based analysis of DTI indices did not reveal any differences between the older adults with cognitive complaints (CC) and healthy controls (HC). [43] This result was consistent with ours. In addition, they conducted another analysis which used parahippocampal region of interest (ROI). In this analysis, the CC group showed diffusivity values that were intermediate to the MCI and HC groups. However, the CC group also had significantly lower memory scores (Wechsler Memory Scale-III, Logical Memory delay recall) than did the HC group. Therefore, the CC subjects in their study had objective memory impairment, while SCI subjects in our study showed no objective memory impairment. This difference may explain the discrepancy between the results of these studies.

In contrast to those previous studies, we could not observe any differences between SCI and NC by DTI analyses or structural neuroimaging. Although it is difficult to collect and assess SCI subjects, we specifically classified people with awareness of poor memory function as SCI or amnestic MCI using cognitive tests and clinical interviews. This may explain why our results differed from other reports investigating SCI. In addition, like other previous reports, if we include subjects with “awareness of poor memory” and normal range MMSE score as SCI, most of our MCI subjects would be diagnosed as SCI. Many amnestic MCI patients are aware of their memory deficits [44]. As mentioned previously, our SCI subjects met the proposed Reisberg criteria for primary idiopathic subjective cognitive impairment [3]. In addition, we did not advertise for the current study, and the SCI subjects voluntarily visited our hospital for consultation on memory deficit.

In future studies, besides CSF biomarkers and amyloid PET examination, it might be of greater benefit to perform functional brain imaging such as functional MRI and near infrared spectroscopy (NIRS) than structural or diffusion MRI. Alternatively, because the current study and previous MRI studies of SCI were conducted using 1.5-T MRI scanners, the use of more than 3-T MRI machines, diffusion tensor or kurtosis imaging may be more useful for an assessment of the very early disease stage [45]. Some of the previous SCI studies investigated APOE genotype and showed that the ε4 genotype but not the non-ε4 genotype was related to future cognitive decline and brain atrophy [37], [41]. Combining those biomarkers and neuroimaging might also be useful for gaining a better understanding of the early pathological changes of Alzheimer's disease.

In conclusion, there were no significant differences between SCI and NC in any measurement. We also observed that AD and MCI subjects had almost the same extent of white matter and atrophic changes. Our results mean that DTI or volumetric analysis of SCI cannot show the early pathological changes. However, our results were consistent with the hypothetical model of dynamic biomarkers of Alzheimer's disease [15] [32].

## Supporting Information

Figure S1 **Differences in fractional anisotropy between Alzheimer's disease and mild cognitive impairment by adding the medial temporal volume as a covariate.**
(TIF)

Figure S2 **Differences in mean diffusivity between Alzheimer's disease and mild cognitive impairment by adding the medial temporal volume as a covariate.**
(TIF)

Table S1 **Voxel-based analysis data of fractional anisotropy among the groups.** R indicates right; L, left; NC, normal control; SCI, subjective cognitive impairment; MCI, mild cognitive impairment; AD, Alzheimer's disease. p-values adjusted for search volume.
(XLS)

Table S2 **Voxel based analysis data of mean diffusivity among the groups.** R indicates right; L, left; NC, normal control; SCI, subjective cognitive impairment; MCI, mild cognitive impairment; AD, Alzheimer's disease. p-values adjusted for search volume.
(XLS)

Table S3 **Voxel based analysis data of grey matter volume among the groups.** R indicates right; L, left; NC, normal control; SCI, subjective cognitive impairment; MCI, mild cognitive impairment; AD, Alzheimer's disease. p-values adjusted for search volume.
(XLS)

## References

[1] Reisberg B, Gauthier S (2008) Current evidence for subjective cognitive impairment (SCI) as the pre-mild cognitive impairment (MCI) stage of subsequently manifest Alzheimer's disease. Int Psychogeriatr 20: 1–16. 18072981 10.1017/S1041610207006412

[2] Wilson RS, Leurgans SE, Boyle PA, Bennett DA (2011) Cognitive decline in prodromal Alzheimer disease and mild cognitive impairment. Archives of neurology 68: 351–356. 21403020 10.1001/archneurol.2011.31 PMC3100533

[3] Reisberg B, Prichep L, Mosconi L, John ER, Glodzik-Sobanska L, et al (2008) The pre-mild cognitive impairment, subjective cognitive impairment stage of Alzheimer's disease. Alzheimers Dement 4: S98–S108. 18632010 10.1016/j.jalz.2007.11.017

[4] Jessen F, Wiese B, Bachmann C, Eifflaender-Gorfer S, Haller F, et al (2010) Prediction of dementia by subjective memory impairment: effects of severity and temporal association with cognitive impairment. Arch Gen Psychiatry 67: 414–422. 20368517 10.1001/archgenpsychiatry.2010.30

[5] Petersen RC, Thomas RG, Grundman M, Bennett D, Doody R, et al (2005) Vitamin E and donepezil for the treatment of mild cognitive impairment. N Engl J Med 352: 2379–2388. 15829527 10.1056/NEJMoa050151

[6] Luis CA, Loewenstein DA, Acevedo A, Barker WW, Duara R (2003) Mild cognitive impairment: directions for future research. Neurology 61: 438–444. 12939414 10.1212/01.wnl.0000080366.90234.7f

[7] Petersen RC (2004) Mild cognitive impairment as a diagnostic entity. J Intern Med 256: 183–194. 15324362 10.1111/j.1365-2796.2004.01388.x

[8] Petersen RC, Stevens JC, Ganguli M, Tangalos EG, Cummings JL, et al (2001) Practice parameter: early detection of dementia: mild cognitive impairment (an evidence-based review). Report of the Quality Standards Subcommittee of the American Academy of Neurology. Neurology 56: 1133–1142. 11342677 10.1212/wnl.56.9.1133

[9] Petersen RC, Doody R, Kurz A, Mohs RC, Morris JC, et al (2001) Current concepts in mild cognitive impairment. Arch Neurol 58: 1985–1992. 11735772 10.1001/archneur.58.12.1985

[10] Lopez OL, Jagust WJ, DeKosky ST, Becker JT, Fitzpatrick A, et al (2003) Prevalence and classification of mild cognitive impairment in the Cardiovascular Health Study Cognition Study: part 1. Arch Neurol 60: 1385–1389. 14568808 10.1001/archneur.60.10.1385

[11] Ganguli M, Dodge HH, Shen C, DeKosky ST (2004) Mild cognitive impairment, amnestic type: an epidemiologic study. Neurology 63: 115–121. 15249620 10.1212/01.wnl.0000132523.27540.81

[12] Grundman M, Petersen RC, Ferris SH, Thomas RG, Aisen PS, et al (2004) Mild cognitive impairment can be distinguished from Alzheimer disease and normal aging for clinical trials. Arch Neurol 61: 59–66. 14732621 10.1001/archneur.61.1.59

[13] Harwood DG, Sultzer DL, Wheatley MV (2000) Impaired insight in Alzheimer disease: association with cognitive deficits, psychiatric symptoms, and behavioral disturbances. Neuropsychiatry, neuropsychology, and behavioral neurology 13: 83–88. 10780626

[14] Galeone F, Pappalardo S (2011) Chieffi S, Iavarone A, Carlomagno S (2011) Anosognosia for memory deficit in amnestic mild cognitive impairment and Alzheimer's disease. International journal of geriatric psychiatry 26: 695–701. 21495076 10.1002/gps.2583

[15] Jack CR Jr, Knopman DS, Jagust WJ, Shaw LM, Aisen PS, et al (2010) Hypothetical model of dynamic biomarkers of the Alzheimer's pathological cascade. Lancet neurology 9: 119–128. 20083042 10.1016/S1474-4422(09)70299-6 PMC2819840

[16] Bateman RJ, Xiong C, Benzinger TL, Fagan AM, Goate A, et al (2012) Clinical and biomarker changes in dominantly inherited Alzheimer's disease. The New England journal of medicine 367: 795–804. 22784036 10.1056/NEJMoa1202753 PMC3474597

[17] Moonis M, Swearer JM, Dayaw MP, St George-Hyslop P, Rogaeva E, et al (2005) Familial Alzheimer disease: decreases in CSF Abeta42 levels precede cognitive decline. Neurology 65: 323–325. 16043812 10.1212/01.wnl.0000171397.32851.bc

[18] Ringman JM, Younkin SG, Pratico D, Seltzer W, Cole GM, et al (2008) Biochemical markers in persons with preclinical familial Alzheimer disease. Neurology 71: 85–92. 18509095 10.1212/01.wnl.0000303973.71803.81 PMC12435649

[19] DeKosky ST, Marek K (2003) Looking backward to move forward: early detection of neurodegenerative disorders. Science 302: 830–834. 14593169 10.1126/science.1090349

[20] Cummings JL, Doody R, Clark C (2007) Disease-modifying therapies for Alzheimer disease: challenges to early intervention. Neurology 69: 1622–1634. 17938373 10.1212/01.wnl.0000295996.54210.69

[21] Selnes P, Aarsland D, Bjornerud A, Gjerstad L, Wallin A, et al (2013) Diffusion tensor imaging surpasses cerebrospinal fluid as predictor of cognitive decline and medial temporal lobe atrophy in subjective cognitive impairment and mild cognitive impairment. J Alzheimers Dis 33: 723–736. 23186987 10.3233/JAD-2012-121603

[22] Snitz BE, Weissfeld LA, Lopez OL, Kuller LH, Saxton J, et al (2013) Cognitive trajectories associated with beta-amyloid deposition in the oldest-old without dementia. Neurology 80: 1378–1384. 23516317 10.1212/WNL.0b013e31828c2fc8 PMC3662268

[23] van Norden AG, Fick WF, de Laat KF, van Uden IW, van Oudheusden LJ, et al (2008) Subjective cognitive failures and hippocampal volume in elderly with white matter lesions. Neurology 71: 1152–1159. 18838662 10.1212/01.wnl.0000327564.44819.49

[24] Striepens N, Scheef L, Wind A, Popp J, Spottke A, et al (2010) Volume loss of the medial temporal lobe structures in subjective memory impairment. Dement Geriatr Cogn Disord 29: 75–81. 20110703 10.1159/000264630

[25] McKhann G, Drachman D, Folstein M, Katzman R, Price D, et al (1984) Clinical diagnosis of Alzheimer's disease: report of the NINCDS-ADRDA Work Group under the auspices of Department of Health and Human Services Task Force on Alzheimer's Disease. Neurology 34: 939–944. 6610841 10.1212/wnl.34.7.939

[26] Gomar JJ, Bobes-Bascaran MT, Conejero-Goldberg C, Davies P, Goldberg TE (2011) Utility of combinations of biomarkers, cognitive markers, and risk factors to predict conversion from mild cognitive impairment to Alzheimer disease in patients in the Alzheimer's disease neuroimaging initiative. Archives of general psychiatry 68: 961–969. 21893661 10.1001/archgenpsychiatry.2011.96

[27] Petersen RC, Aisen PS, Beckett LA, Donohue MC, Gamst AC, et al (2010) Alzheimer's Disease Neuroimaging Initiative (ADNI): clinical characterization. Neurology 74: 201–209. 20042704 10.1212/WNL.0b013e3181cb3e25 PMC2809036

[28] Fazekas F, Chawluk JB, Alavi A, Hurtig HI, Zimmerman RA (1987) MR signal abnormalities at 1.5 T in Alzheimer's dementia and normal aging. AJR American journal of roentgenology 149: 351–356. 3496763 10.2214/ajr.149.2.351

[29] Reisberg B, Ferris SH, de Leon MJ, Crook T (1982) The Global Deterioration Scale for assessment of primary degenerative dementia. The American journal of psychiatry 139: 1136–1139. 7114305 10.1176/ajp.139.9.1136

[30] Reisberg B, Ferris SH, de Leon MJ, Crook T (1988) Global Deterioration Scale (GDS). Psychopharmacology bulletin 24: 661–663. 3249768

[31] Ashburner J, Friston KJ (2005) Unified segmentation. NeuroImage 26: 839–851. 15955494 10.1016/j.neuroimage.2005.02.018

[32] Jack CR Jr, Knopman DS, Jagust WJ, Petersen RC, Weiner MW, et al (2013) Tracking pathophysiological processes in Alzheimer's disease: an updated hypothetical model of dynamic biomarkers. Lancet neurology 12: 207–216. 23332364 10.1016/S1474-4422(12)70291-0 PMC3622225

[33] Kiuchi K, Morikawa M, Taoka T, Nagashima T, Yamauchi T, et al (2009) Abnormalities of the uncinate fasciculus and posterior cingulate fasciculus in mild cognitive impairment and early Alzheimer's disease: a diffusion tensor tractography study. Brain Res 1287: 184–191. 19559010 10.1016/j.brainres.2009.06.052

[34] Wolkin A, Rusinek H, Vaid G, Arena L, Lafargue T, et al (1998) Structural magnetic resonance image averaging in schizophrenia. The American journal of psychiatry 155: 1064–1073. 9699695 10.1176/ajp.155.8.1064

[35] Kubicki M, Shenton ME, Salisbury DF, Hirayasu Y, Kasai K, et al (2002) Voxel-based morphometric analysis of gray matter in first episode schizophrenia. NeuroImage 17: 1711–1719. 12498745 10.1006/nimg.2002.1296 PMC2845166

[36] Kennedy KM, Erickson KI, Rodrigue KM, Voss MW, Colcombe SJ, et al (2009) Age-related differences in regional brain volumes: a comparison of optimized voxel-based morphometry to manual volumetry. Neurobiology of aging 30: 1657–1676. 18276037 10.1016/j.neurobiolaging.2007.12.020 PMC2756236

[37] Stewart R, Godin O, Crivello F, Maillard P, Mazoyer B, et al (2011) Longitudinal neuroimaging correlates of subjective memory impairment: 4-year prospective community study. The British journal of psychiatry: the journal of mental science 198: 199–205. 21357878 10.1192/bjp.bp.110.078683

[38] Petersen RC (2004) Mild cognitive impairment as a diagnostic entity. Journal of internal medicine 256: 183–194. 15324362 10.1111/j.1365-2796.2004.01388.x

[39] Gauthier S, Reisberg B, Zaudig M, Petersen RC, Ritchie K, et al (2006) Mild cognitive impairment. Lancet 367: 1262–1270. 16631882 10.1016/S0140-6736(06)68542-5

[40] Selnes P, Fjell AM, Gjerstad L, Bjornerud A, Wallin A, et al (2012) White matter imaging changes in subjective and mild cognitive impairment. Alzheimer's & dementia: the journal of the Alzheimer's Association 8: S112–121. 10.1016/j.jalz.2011.07.001 23021621

[41] Grambaite R, Stenset V, Reinvang I, Walhovd KB, Fjell AM, et al (2010) White matter diffusivity predicts memory in patients with subjective and mild cognitive impairment and normal CSF total tau levels. Journal of the International Neuropsychological Society: JINS 16: 58–69. 19835655 10.1017/S1355617709990932

[42] Stenset V, Bjornerud A, Fjell AM, Walhovd KB, Hofoss D, et al (2011) Cingulum fiber diffusivity and CSF T-tau in patients with subjective and mild cognitive impairment. Neurobiology of aging 32: 581–589. 19428143 10.1016/j.neurobiolaging.2009.04.014

[43] Wang Y, West JD, Flashman LA, Wishart HA, Santulli RB, et al (2012) Selective changes in white matter integrity in MCI and older adults with cognitive complaints. Biochimica et Biophysica Acta 1822: 423–430. 21867750 10.1016/j.bbadis.2011.08.002 PMC3235544

[44] Vogel A, Hasselbalch SG, Gade A, Ziebell M, Waldemar G (2005) Cognitive and functional neuroimaging correlate for anosognosia in mild cognitive impairment and Alzheimer's disease. International journal of geriatric psychiatry 20: 238–246. 15717342 10.1002/gps.1272

[45] Kamagata K, Tomiyama H, Motoi Y, Kano M, Abe O, et al (2013) Diffusional kurtosis imaging of cingulate fibers in Parkinson disease: comparison with conventional diffusion tensor imaging. Magnetic resonance imaging 31: 1501–1506. 23895870 10.1016/j.mri.2013.06.009

