# Supplementary figures and images for: Gray and White Matter Changes in Subjective Cognitive Impairment, Amnestic Mild Cognitive Impairment and Alzheimer's Disease: A Voxel-Based Analysis Study
Source: PLoS One. 2014 Aug 5;9(8):e104007. doi: 10.1371/journal.pone.0104007 (PMC4122459; doi:10.1371/journal.pone.0104007)

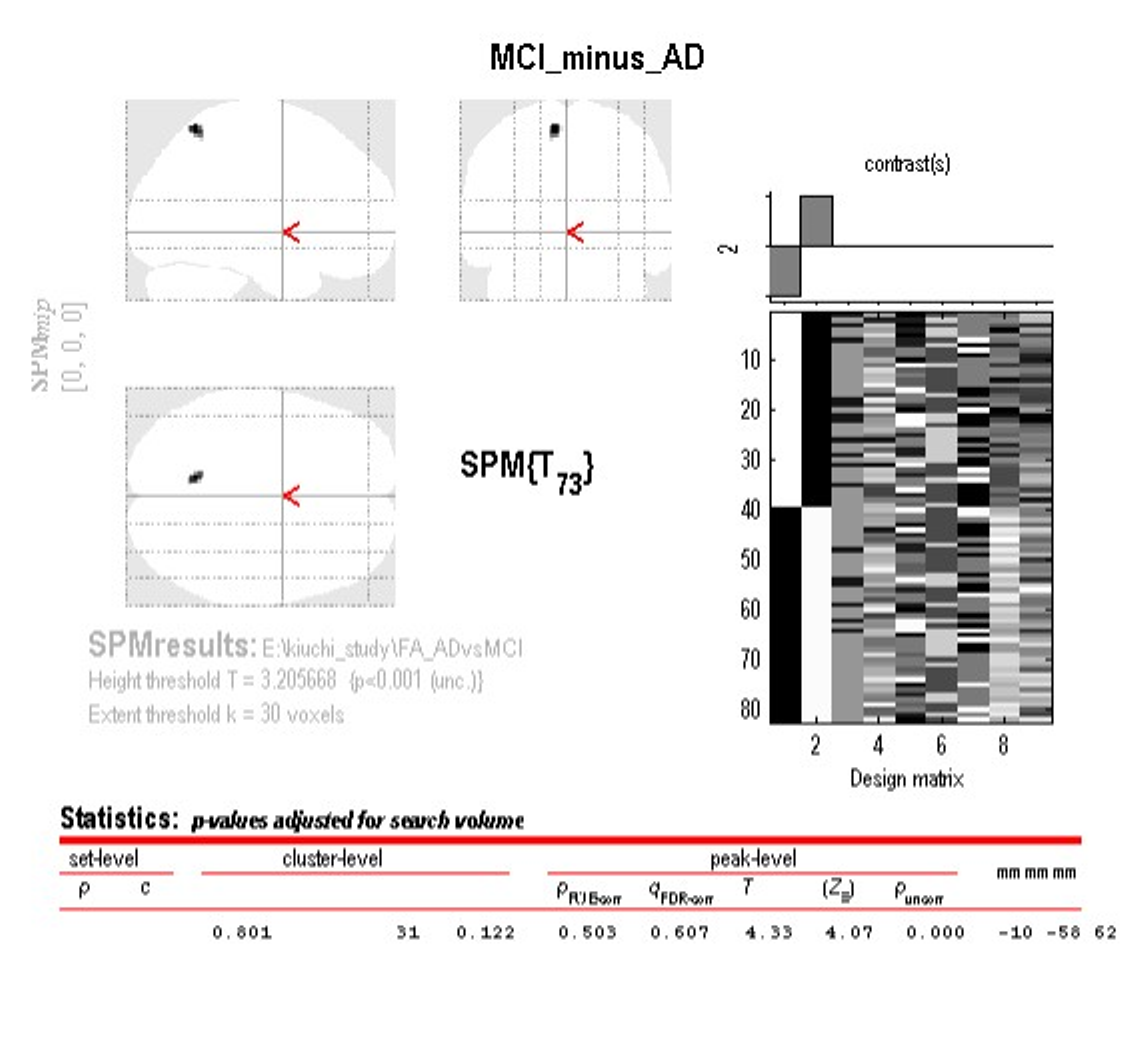

Supplement: Figure S1 — Differences in fractional anisotropy between Alzheimer's disease and mild cognitive impairment by adding the medial temporal volume as a covariate. (TIF) [file pone.0104007.s001.tif]

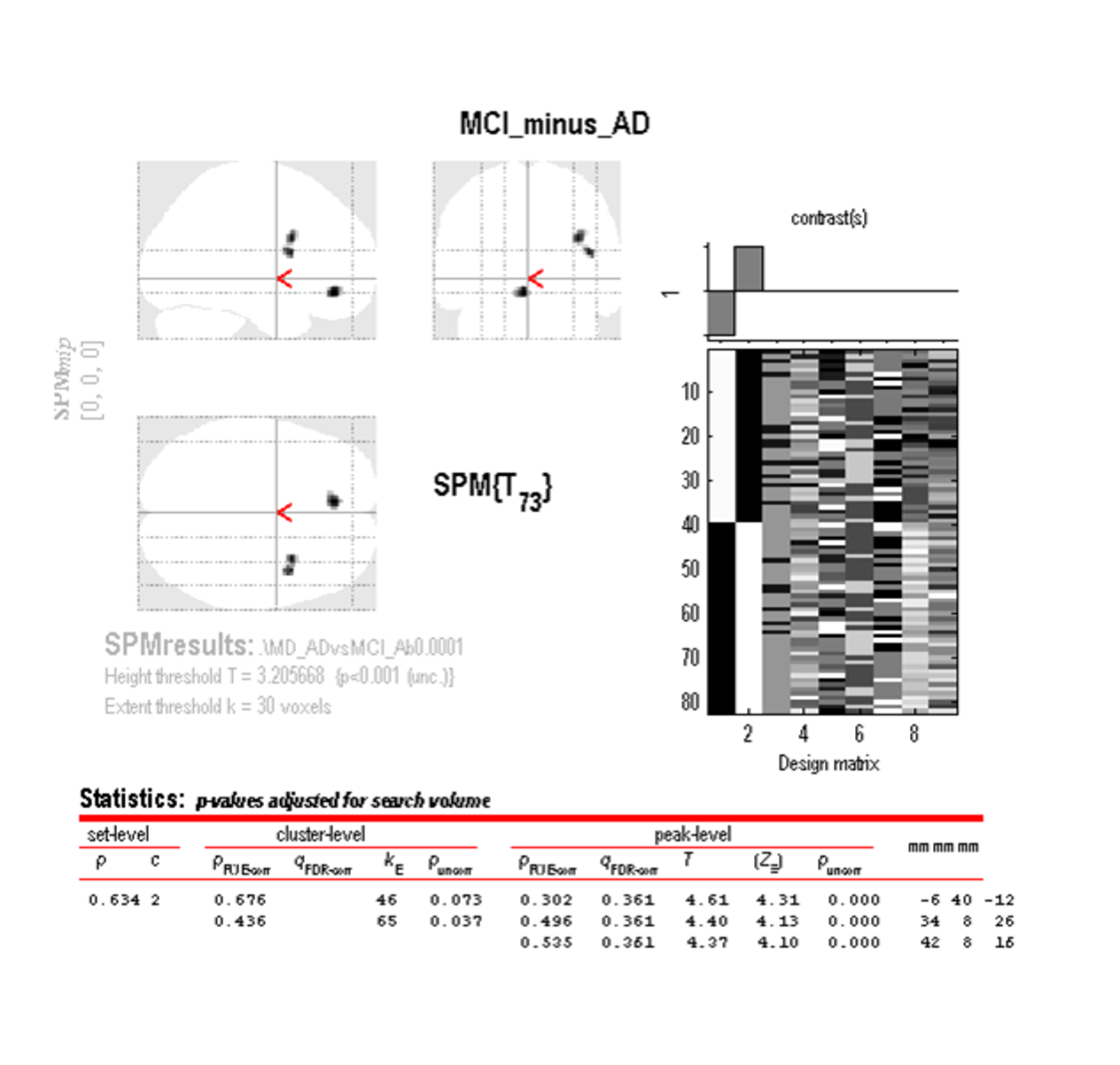

Supplement: Figure S2 — Differences in mean diffusivity between Alzheimer's disease and mild cognitive impairment by adding the medial temporal volume as a covariate. (TIF) [file pone.0104007.s002.tif]
